# Supplementary material for: Chromothripsis during telomere crisis is independent of NHEJ, and consistent with a replicative origin
Source: Genome Res. 2019 May;29(5):737–49. doi: 10.1101/gr.240705.118 (PMC6499312; doi:10.1101/gr.240705.118)
Supplement: Supplemental Material [file supp_gr.240705.118_Supplemental_file_1.zip › contigs/annotated_contigs/DB56/contig.2.DB56_length_735_mean_cov_9.59183673469.docx]

**DB56_length_735_mean_cov_9.59183673469**

```
AAATTTTAATAAAAGCCTGAAAGATATTTCACCATCAATTTCTGAGGGTCTACTCAGGGCAGACGGTGCAGTGTAAGTTGTAAGGTTAA
 >chr2:3208122-3208510 + E=1e-215
TCAATCACAATTTTTGGTTTCCTGGGGACATGGAGACAGCACTCAGCAAGAACTCCACAGGGTGTGATGCATGCAGTAATCTGTTTTTC

GACATTAAAGAAGAATATTCAGTGCTTCCTGGTCCAAAAGGAATGATTAAAGGCGCCCACAAAACTTTCAAGGGAGATGTTACTAATGC

CAGAATGTGAACAAAAGAATACACCTCCTAACGAGCAAAATGACGTGCCTGGCATCGTCTTTAGCAGTTCATGTGGAGCATATCTAAGA

AGAGTCTCAGCGGAAGATTTTGAGAGACAAA|A|CTGCTCCATGTTCTGTTCTTTAATTTCTGAGCAAATCAACTATCGTTCCTTTTTA
                                >chr2:3116391-3116734 - E=6e-185
AAATGTTCTTATGCTCCCGTTGTTTCTTTCTGAGTTGCTGTTTTGTTTTGTTTGCGTGTGCTGGGTTTGTCTCTGTGTTGGAGATCTCC

CCCATGTGCCTGGTGATAATGGGCAAGTTCGCCCACGTTTGTGAGCGTTAGCACCTACTACAGGCTTTGTGTGCACGGGTGGGGCTTGC

TGCGTTGTGAGCTTTGTTCTAGGATACTGGGAAGGGGAGCCAATGGTTGCAGTGTGAAATTCCCAACTGACAGAATCTGCAGGTCTTTT

TCCTTGGGCTAGTCAATTTCCTCAG
```
